# Antifungal Activity and Biochemical Mechanisms of Artemisinin Against the Phytopathogen *Sclerotinia sclerotiorum*

**DOI:** 10.3390/ijms27083422

**Published:** 2026-04-10

**Authors:** Yuxin Zhao, Xin Li, Hai-Ning Lyu, Jingjing Liao, Jiale Xing, Xin Zhao, Qian Zhang, Huanling Yang, Shuyu Li, Junzhe Zhang, Qiaoli Shi, Chengchao Xu, Xin Chai

**Affiliations:** 1 State Key Laboratory for Quality Ensurance and Sustainable Use of Dao-di Herbs, Artemisinin Research Center, Institute of Chinese Materia Medica, China Academy of Chinese Medical Sciences, Beijing 100700, China; 2 Department of Nephrology, Shenzhen Clinical Research Centre for Geriatrics, Shenzhen People’s Hospital (The Second Clinical Medical College, Jinan University; The First Affiliated Hospital, Southern University of Science and Technology), Shenzhen 518020, China; 3 Graduate School of China Academy of Chinese Medical Sciences, Suzhou 215105, China

**Keywords:** artemisinin, *Sclerotinia sclerotiorum*, antifungal activity, proteomic analysis

## Abstract

*Sclerotinia sclerotiorum* (Lib.) de Bary is a globally distributed necrotrophic fungal pathogen capable of infecting a wide range of crops. While conventional chemical fungicides offer effective control, their long-term use leads to increased fungicide resistance and poses risks to the environment and human health due to pesticide residues, underscoring the urgent need to develop novel fungicides. Artemisinin, first identified in *Artemisia annua*, is renowned for its antimalarial activity. Here, we demonstrate that artemisinin exhibites effective antifungal activity against *Sclerotinia sclerotiorum* with an EC_50_ value of 0.1 mg/mL. Treatment with artemisinin caused the mycelia surface to collapse and shrivel, accompanied by enhanced membrane permeability. Pretreating *Brassica napus* and *Arabidopsis* leaves with artemisinin increased resistance to *S. sclerotiorum* infection. Proteomic analysis revealed that artemisinin treatment markedly downregulated the expression of key functional proteins in *S. sclerotiorum*, including enzymes involved in oxalic acid biosynthesis, cell wall-associated proteins, and secreted proteins. In conclusion, artemisinin exhibits notable inhibitory effects against *S. sclerotiorum* and may hold potential for development as a novel fungicide.

## 1. Introduction

*Sclerotinia sclerotiorum* (Lib.) de Bary is a globally distributed necrotrophic fungal pathogen that infects plants by killing host cells and absorbing nutrients through its hyphae, ultimately causing plant death. The resulting disease, known as Sclerotinia stem rot (also called white mold or stem rot), affects a broad host range of over 700 plant species, including many economically important crops, and causes severe economic losses worldwide [1,2,3]. Owing to its broad host range and the limited availability of genetically resistant cultivars, chemical fungicides remain the primary strategy for disease control. However, the extensive and long-term application of fungicides has contributed to the development of resistance in *S. sclerotiorum*. For example, benzimidazole fungicide carbendazim has been used in China for nearly 40 years [4], but carbendazim-resistant populations have emerged, markedly reducing its efficacy [5,6]. In the past decade, the dicarboximide fungicides dimetachlone and iprodione have also been applied [7]; however, dimetachlone-resistant strains have recently been reported in China [8,9]. While pesticide residues pose increasing risks to environmental and human health [10,11,12,13]. Therefore, the development of novel, natural, and environmentally friendly fungicides is urgent.

Compared to synthetic compounds, natural products offer several advantages including structural diversity, relatively low toxicity, and a broad spectrum of biological activities [14,15,16]. Many plant-derived natural products play important roles in adaptation to biotic and abiotic stresses. These compounds help plants resist adverse environmental conditions and pathogen infections. In recent years, there has been a significant increase in reports on natural products inhibiting plant pathogens. For instance, 4-hydroxybenzoic acid and vanillic acid isolated from *Sedum* root extracts were reported to significantly alleviate leaf yellowing symptoms in tomato caused by *Pseudomonas syringae* pv. tomato DC3000 [17]. Similarly, pristimerin extracted from *Pristimera* species has been shown to inhibit the growth of *S. sclerotiorum* [18].

Artemisinin, a sesquiterpene lactone derived from *Artemisia annua*, is well known for its antimalarial activity [19,20,21,22]. Beyond its antimalarial effects, artemisinin has also been reported to possess anticancer effects against various types of cancers [23,24,25,26]. Artemisinin is stored in glandular trichomes on the surface of the leaves and stems [27] and on the corolla and receptacles of the florets [28]. Emerging evidence suggests that artemisinin also plays ecological roles in plant defense, including deterring herbivorous insects and inhibiting pathogen infection [29,30]. For example, Maggi et al. (2005) reported that artemisinin exhibits feeding deterrent activity against *Epilachna paenulata* and *Spodoptera eridania* [31]. Moreover, artemisinin has shown inhibitory effects against several fungal pathogens, including *Candida albicans*, *Aspergillus niger*, *Fusarium* spp., *Trichoderma viride*, and *Aspergillus flavus* [32,33].

In the present study, we demonstrate that artemisinin effectively suppresses the growth of *S. sclerotiorum*. Artemisinin treatment resulted in marked morphological alterations in the mycelia, including surface collapse and shrinkage, accompanied by increased membrane permeability. Furthermore, pretreatment of *Brassica napus* and *Arabidopsis thaliana* leaves enhanced resistance to *S. sclerotiorum* infection. Proteomic analysis revealed that artemisinin significantly downregulated proteins associated with cellular growth and development, as well as multiple pathogenicity-related factors, including enzymes involved in oxalate metabolism, plant cell wall degradation, and other virulence-associated processes. Collectively, these findings highlight the potent antifungal activity of artemisinin against *S. sclerotiorum* and suggest its potential as a promising eco-friendly candidate for agricultural fungicide development.

## 2. Results

### 2.1. Artemisinin Inhibits the Growth of S. sclerotiorum in a Concentration-Dependent Manner

**Figure 1:**
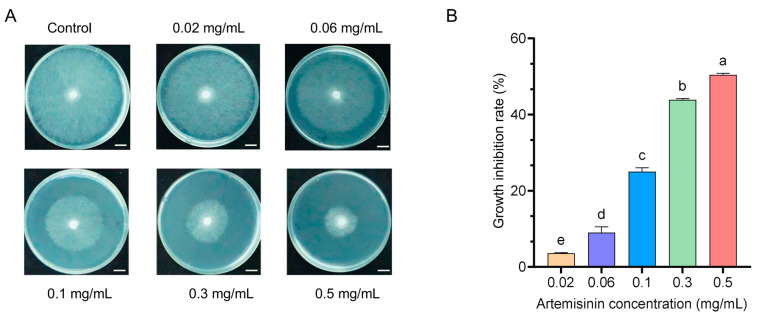
Artemisinin inhibits the growth of *S. sclerotiorum.* (**A**) Growth of *S. sclerotiorum* was assessed on PDA plates amended with artemisinin at final concentrations ranging from 0 to 0.5 mg/mL. The control group received 0.2% DMSO. Scale bar, 1 cm. (**B**) Quantitative analysis of the inhibitory effect of varying concentrations of artemisinin on *S. sclerotiorum*. Growth inhibition rates were calculated relative to control colonies. Error bars, means ± SD (*n* = 3). Different lowercase letters above the bars indicate statistically significant differences between groups (one-way ANOVA followed by Tukey’s multiple comparison test).

To evaluate the antifungal activity of artemisinin against *S. sclerotiorum,* culture media supplemented with varying concentrations of artemisinin were prepared. Mycelia taken from freshly cultured *S. sclerotiorum* were inoculated. After three days of incubation, the mycelia of the control group had fully covered the plates. The colony diameters of both the control and treatment groups were measured, and the growth curve, inhibition rate, and EC50 were calculated. The results show that artemisinin inhibited mycelial growth in a dose-dependent manner (Figure 1A and Figure S1), with the inhibitory rate increasing as the artemisinin concentration increased (Figure 1B). The half-maximal effective concentration (EC50) was determined to be 0.1 mg/mL (Table S1). These results indicate that artemisinin effectively inhibits the growth of *S. sclerotiorum* in a concentration-dependent manner.

### 2.2. Artemisinin Disrupts the Mycelial Morphology of S. sclerotiorum

**Figure 2:**
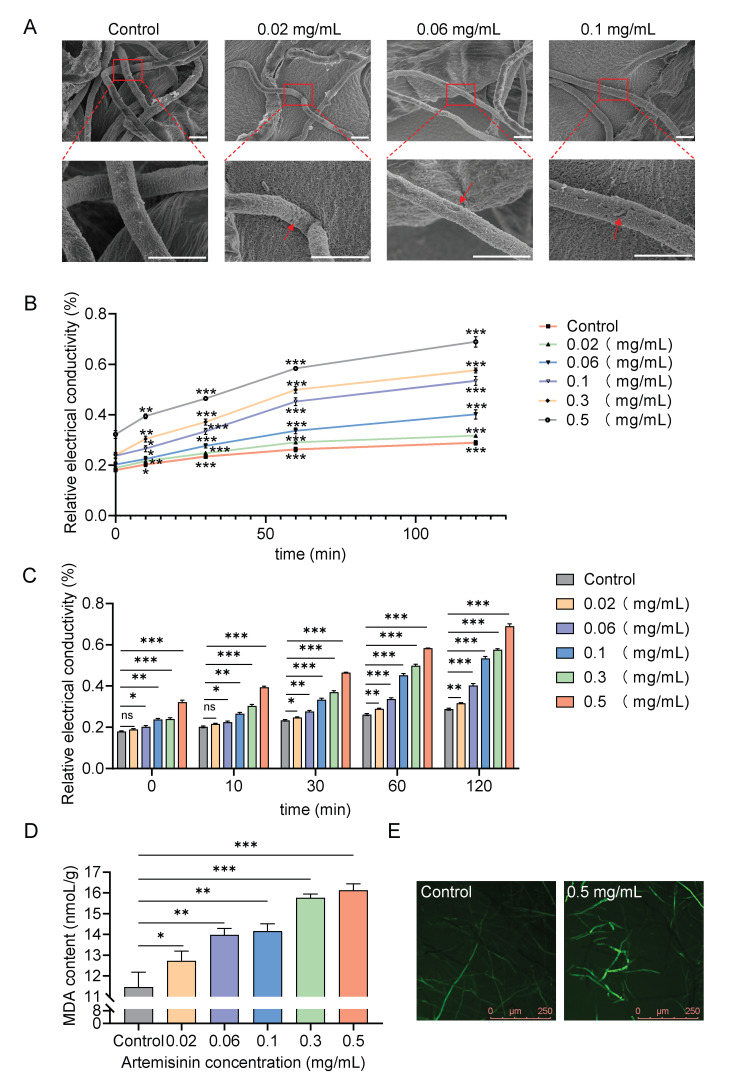
Artemisinin disrupts mycelial morphology of *S. sclerotiorum.* (**A**) Scanning electron microscopy images of *S. sclerotiorum* mycelial morphology after treatment with the indicated concentrations of artemisinin. Red arrows indicate the sites of depression and shriveling. Scale bars, 5 μm. (**B**) Time-course analysis of conductivity in *S. sclerotiorum* exposed to varying artemisinin concentrations. Data are presented as means ± SD (*n* = 3). Asterisks indicate significant differences compared with the corresponding 0 min time point within the control and treatment groups, respectively (two-tailed unpaired Student’s *t*-test, * *p* < 0.05, ** *p* < 0.01, *** *p* < 0.001). (**C**) Conductivity of *S. sclerotiorum* measured at different time points post-treatment with the indicated artemisinin concentrations. Error bars represent means ± SD (*n* = 3). Asterisks indicate statistically significant differences between each artemisinin-treated group and the control group at the same time point (two-tailed unpaired Student’s *t*-test, ns: not significant (*p* ≥ 0.05), * *p* < 0.05, ** *p* < 0.01, *** *p* < 0.001). (**D**) Measurement of MDA content in control and artemisinin-treated groups. Error bars represent means ± SD (*n* = 3). Asterisks indicate statistically significant differences between each artemisinin-treated group and the control group (two-tailed unpaired Student’s *t*-test, * *p* < 0.05, ** *p* < 0.01, *** *p* < 0.001). (**E**) Fluorescence microscopic observation of ROS in mycelia of control and 0.5 mg/mL artemisinin-treated groups.

To further investigate the effects of artemisinin on *S. sclerotiorum*, we employed scanning electron microscopy (SEM) to examine the mycelial morphology following treatment with various concentrations of artemisinin. As shown in Figure 2A, mycelia from the control group exhibited normal morphology with smooth surfaces. Notably, treatment with 0.02 mg/mL artemisinin induced initial abnormalities on the mycelia surface, including collapse and shriveling. With increasing artemisinin concentrations, these morphological distortions became progressively more severe, indicating dose-dependent structural damage.

To determine whether artemisinin affects membrane integrity, cell membrane permeability was evaluated by measuring electrolyte leakage. We cultured the mycelia and treated them with different concentrations of artemisinin for 36 h. After treatment, the mycelia were collected and placed in distilled water. The electrical conductivity of the suspension was monitored over time. As shown in Figure 2B,C, the conductivity increased progressively after treatment, indicating enhanced electrolyte leakage from the mycelia. The magnitude of the increase in conductivity correlated with artemisinin concentration. These results suggest that artemisinin compromises cell membrane integrity, leading to increased permeability and cellular damage.

Damage to the cell membrane induces the accumulation of reactive oxygen species (ROS) and triggers lipid peroxidation. Malondialdehyde (MDA) is a widely used indicator of membrane lipid peroxidation [34]; thus, the levels of ROS and MDA can effectively reflect the degree of cell membrane damage. In this study, ROS levels were detected using DCFH-DA staining, while MDA content was measured using a commercial kit. The results showed that artemisinin treatment increased ROS accumulation in mycelia (Figure 2E). Moreover, MDA content increased in a concentration-dependent manner with artemisinin treatment (Figure 2D). Collectively, these findings indicate that artemisinin disrupts the cell membrane structure of *S. sclerotiorum*.

### 2.3. Artemisinin Pretreatment Enhances Resistance to S. sclerotiorum in Brassica napus and Arabidopsis

**Figure 3:**
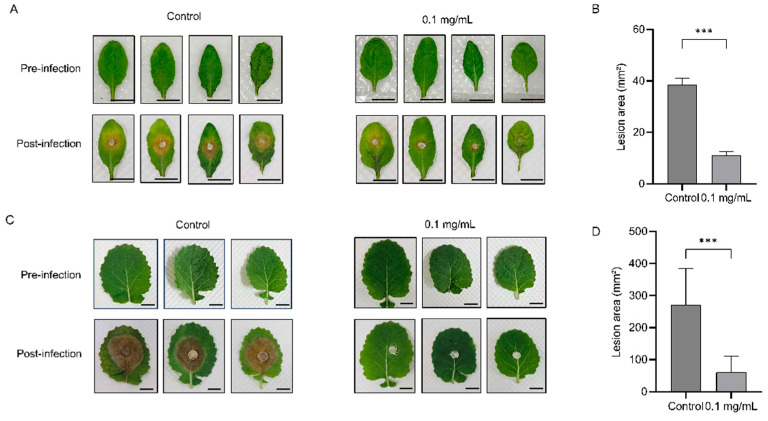
Protective effects of artemisinin against *S. sclerotiorum* infection on *Brassica napus* and *Arabidopsis* leaves. (**A**) *Arabidopsis* leaves were treated with 0.1 mg/mL artemisinin, with 0.2% DMSO serving as the control. After 24 h, the leaves were inoculated with *S. sclerotiorum* mycelial plugs and photographed two days post-inoculation. Scale bar, 1 cm. (**B**) Lesion areas in control and artemisinin-treated *Arabidopsis* leaves were measured using ImageJ (version 1.54g) software. Data are presented as the mean of ten replicates per treatment. Error bars, means ± SD (*n* = 10). Asterisks indicate statistically significant differences compared with the control group (two-tailed unpaired Student’s *t*-test, *** *p* < 0.001). (**C**) *B. napus* leaves were treated with artemisinin at a concentration of 0.1 mg/mL, with 0.2% DMSO serving as the control. After 24 h, the leaves were inoculated with *S. sclerotiorum* mycelial plugs and photographed three days post-inoculation. Scale bar, 1 cm. (**D**) Lesion areas in *B. napus* leaves of control and artemisinin-treated samples were measured using ImageJ software; each treatment mean represents the average of ten replicates. Error bars, means ± SD (*n* = 10). Asterisks indicate statistically significant differences compared with the control group (two-tailed unpaired Student’s *t*-test, *** *p* < 0.001).

*S. sclerotiorum* can infect over 700 plant species, leading to necrosis of plant cells and tissues. Among economically important crops, *Brassica napus* is the most severely affected by *S. sclerotiorum*, posing a significant threat to the security of the oilseed industry. Based on our findings that artemisinin inhibits the growth of *S. sclerotiorum*, we further investigated whether artemisinin could enhance the resistance of *Brassica napus* to this pathogen. We sprayed *Brassica napus* leaves with 0.1 mg/mL artemisinin and then inoculated them with *S. sclerotiorum*. After three days, the lesion areas were examined. The results showed that the leaves treated with artemisinin exhibited significantly smaller lesions, with an approximately 80% reduction in lesion area (Figure 3C,D). A similar protective effect was observed in the model plant *Arabidopsis thaliana*, where artemisinin pretreatment markedly reduced disease symptoms (Figure 3A,B). To assess phytotoxicity, we sprayed artemisinin on *Arabidopsis*, *Nicotiana benthamiana*, and *Brassica napus* seedlings, as well as flowering *Brassica napus* plants. All plants appeared normal after two days, showing no signs of phytotoxicity (Figure S2). These results confirm that artemisinin does not cause host tissue damage at the concentration used, and the observed lesion reduction is attributable to antifungal activity rather than non-specific plant stress. Collectively, these findings suggest that artemisinin has potential for development as a green pesticide for the control of plant pathogen infections.

### 2.4. Effects of Artemisinin on the Proteome of S. sclerotiorum

**Figure 4:**
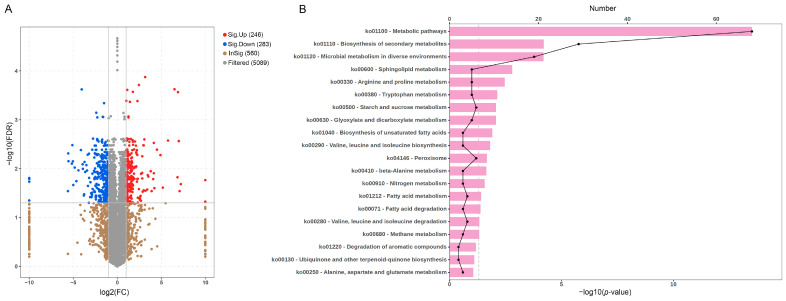
Proteomic analysis of *S. sclerotiorum* following artemisinin treatment. (**A**) Volcano plot showing differentially expressed proteins in *S. sclerotiorum* following treatment with 0.05 mg/mL artemisinin. Differentially expressed proteins were identified using the following thresholds: log_2_(fold change) > 1 and −log_10_(FDR) > 1.3, corresponding to an adjusted *p*-value (FDR) of <0.05. Red dots represent upregulated proteins compared to the control group, while blue dots represent downregulated proteins. (**B**) KEGG enrichment analysis of downregulated proteins after artemisinin treatment.

To investigate the molecular mechanisms underlying artemisinin-mediated growth inhibition, *S. sclerotiorum* was treated with 0.05 mg/mL artemisinin for three days, and mycelia were subjected to quantitative proteomic analysis with three independent biological replicates per group. Principal component analysis (PCA) revealed close clustering among replicates within each group and a clear separation between the control and artemisinin-treated groups, confirming dataset reliability (Figure S3). Proteins with an absolute fold change ≥ 2 and false discovery rate (FDR) < 0.05 (Benjamini–Hochberg corrected *t*-test) were considered significantly changed. Compared with the control group, artemisinin treatment significantly altered the expression of 529 proteins, of which 283 were downregulated and 246 upregulated (Figure 4A). KEGG enrichment analysis (hypergeometric test, Benjamini–Hochberg corrected) identified pathways with FDR < 0.05 as significantly enriched. The downregulated proteins were predominantly enriched in metabolic pathways, biosynthesis of secondary metabolites, and microbial metabolism in diverse environments, along with other key biological processes (Figure 4B and Figure S4).

It is well established that *S. sclerotiorum* causes plant cell death by secreting oxalic acid, plant cell wall-degrading enzymes, and various virulence factors to disrupt host cell structure [35,36,37,38]. Fungi synthesize oxalic acid through three major pathways: the cytoplasmic pathway, where oxaloacetate (from pyruvate carboxylation) is cleaved by OAH to yield oxalate [39,40,41]; the mitochondrial TCA pathway, in which oxaloacetate generated within the cycle is similarly hydrolyzed by OAH [42,43]; and the glyoxysomal glyoxylate (GLOX) pathway, where glyoxylate produced by GLOXDH from citrate is further converted to oxalate [44,45,46]. Synthesized oxalate is then secreted via a dedicated transporter [47].

**Figure 5:**
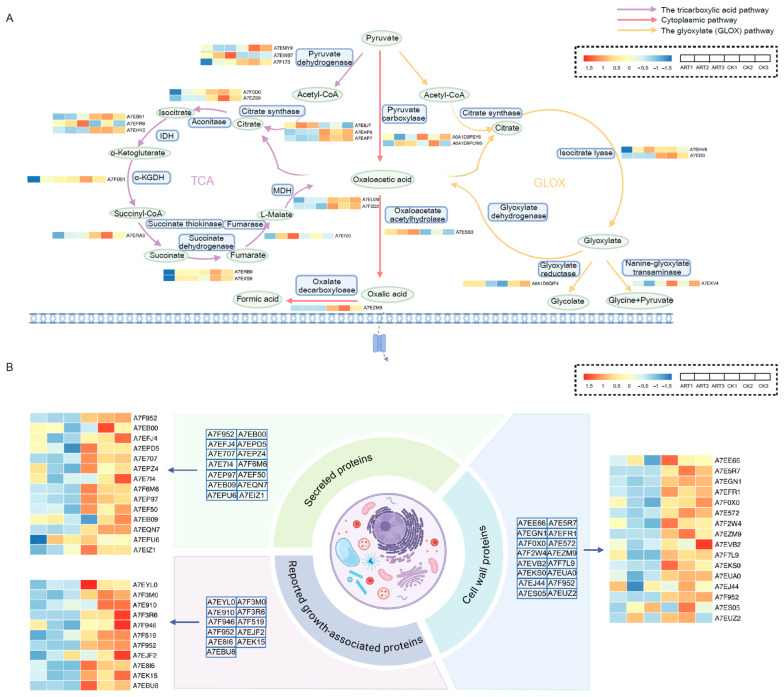
Representative differentially expressed proteins. (**A**) Proteomic profiling of oxalate synthesis-associated proteins in *S. sclerotiorum* in response to artemisinin. Oxalic acid is synthesized via three distinct pathways: the cytoplasmic pathway, the tricarboxylic acid (TCA) cycle, and the glyoxylate (GLOX) pathway. The expression levels of corresponding enzymes identified in the proteomic dataset are visualized in the heatmap. The dashed box in the top right corner contains the schematic diagram corresponding to the heatmap. CK1, CK2, and CK3 denote three biological replicates of the control group, while ART1, ART2, and ART3 represent three replicates of the artemisinin-treated group. Detailed functional descriptions of these enzymes are available in Table S2. (**B**) Expression profiling of cell wall-associated proteins, secreted proteins and growth-related proteins in response to artemisinin treatment. The list of relevant proteins identified in the proteomic dataset is depicted in the schematic diagram, with the corresponding protein heatmap displayed adjacent to the list. ART1, ART2, and ART3 refer to three biological replicates of the artemisinin-treated group, while CK1, CK2, and CK3 represent three replicates of the control group. Detailed functional descriptions of the corresponding proteins are available in the Supporting Information (Tables S3–S5).

**Figure 6:**
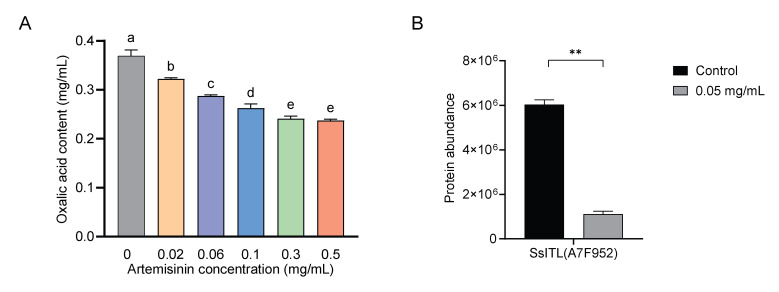
Validation of oxalic acid content and secreted protein expression. (**A**) Oxalic acid content in control and artemisinin treatment groups. Error bars represent means ± SD (*n* = 3). Different lowercase letters above the bars indicate statistically significant differences between groups (one-way ANOVA followed by Tukey’s multiple comparison test). (**B**) Protein levels of SsITL were determined by LC-MS-based proteomic analysis. The bar graph shows the relative abundance of SsITL in mycelia from the control group and the group treated with artemisinin. Data are presented as means ± SD (*n* = 3). Asterisks indicate statistically significant differences compared with the control group (two-tailed unpaired Student’s *t*-test, ** *p* < 0.01).

Our proteomic data revealed that following artemisinin treatment, the majority of enzymes involved in these three pathways are downregulated, while a few specific proteins are upregulated (Figure 5A; Table S2). These results suggest that artemisinin treatment significantly perturbed the oxalate metabolism. To assess whether artemisinin treatment reduces oxalic acid content, the oxalic acid level in mycelia was determined based on the absorbance at 510 nm and calculated using standard curves. As presented in Figure 6A, oxalic acid content was significantly downregulated in a concentration-dependent manner following artemisinin treatment.

Furthermore, we observed a broad downregulation of secreted proteins, and other proteins involved in the growth and development of *S. sclerotiorum* (Tables S3–S5). For instance, virulence factor SsITL (A7F952), which suppresses plant immune responses during early infection [48], was downregulated approximately six-fold (Figure 5B and Figure 6B). Additionally, a significant reduction in several cell wall protein components was detected.

These results indicated that artemisinin reduces the levels of proteins associated with cell wall composition and cell growth in *S. sclerotiorum*, thereby disrupting its cellular structure and inhibiting mycelial development. Furthermore, artemisinin interferes with the biosynthesis of plant-pathogenic factors in *S. sclerotiorum*, including the suppression of oxalate metabolism and a significant reduction in the production of secreted proteins harmful to plants.

## 3. Discussion

*S. sclerotiorum* is a destructive, broad-spectrum plant pathogen. Current control relies mainly on chemical pesticides. Nevertheless, increasing concerns regarding fungicide residues, environmental safety, and resistance development have driven the search for natural products as sustainable alternatives for plant disease control. Artemisinin, a sesquiterpene lactone secondary metabolite produced by *Artemisia annua*, is best known as a powerful antimalarial compound. Recent studies have expanded our understanding of its pharmacological potential, including antitumor, anti-inflammatory, and antimicrobial activities.

In this study, we evaluated the inhibitory effect of artemisinin on *S. sclerotiorum*. The results showed that artemisinin effectively inhibited the growth of *S. sclerotiorum*. Scanning electron microscopy observation revealed that artemisinin treatment caused collapse and shriveling on the hyphal surface. Measurements of electrical conductivity indicated an increase in cell membrane permeability. ROS accumulation and elevated MDA content reflect the extent of cell membrane structural damage. Pre-treatment of plant leaves with artemisinin effectively enhanced their resistance against *S. sclerotiorum* infection. Collectively, these results indicate that artemisinin exerts strong antifungal activity and may serve as a promising eco-friendly candidate for plant disease control.

To investigate the molecular mechanism of artemisinin’s antifungal activity, we first treated *S. sclerotiorum* with artemisinin and then performed proteomic mass spectrometry analysis. The cell wall of *S. sclerotiorum* is essential for preserving cell integrity, safeguarding internal structures, and resisting environmental stress. Importantly, it orchestrates host–pathogen interactions by employing surface sensors to monitor external cues and adhesion proteins to promote tissue adhesion, intercellular communication, and biofilm formation [49,50,51]. Based on the disruption of the cell wall structure observed by scanning electron microscopy, we analyzed the cell wall component proteins identified by mass spectrometry. The results showed that most cell wall proteins were downregulated. In addition, key proteins previously reported to be involved in the growth and development of *S. sclerotiorum*—such as necrosis and ethylene inducing protein 2 and mitogen-activated protein kinases, which influence hyphal growth and sclerotial formation—also decreased. These findings suggest that artemisinin disrupts the cellular structure and inhibits the growth of *S. sclerotiorum* by reducing the synthesis of cell wall component proteins and other growth/development-related proteins.

During the infection of plant tissues, *S. sclerotiorum* secretes oxalic acid, cell wall-degrading enzymes, and toxic proteins that suppress host immune responses. Oxalic acid, a small-molecule organic acid, serves as a virulence factor in many necrotrophic fungal pathogens. It lowers the pH of the surrounding environment, maintaining consistently acidic conditions that favor the growth of *S. sclerotiorum* and enhance the activity of plant cell wall-degrading enzymes. In addition, oxalic acid disrupts plant cell wall integrity by chelating Ca^2+^ from the cell wall, forming insoluble calcium oxalate crystals that block plant vessels and acidify host cells [52,53]. Our proteomic analysis showed that most enzymes involved in the three major oxalate biosynthesis pathways were downregulated following artemisinin treatment, indicating a substantial disruption of oxalate metabolism. Although a few enzymes were upregulated, the overall pattern suggests impaired metabolic homeostasis. The oxalic acid content assay showed that oxalic acid levels were significantly decreased in the artemisinin-treated group. Moreover, multiple plant cell wall–degrading enzymes, including pectinases and cellulases, as well as several secreted effector proteins, were significantly reduced. The coordinated downregulation of oxalate biosynthesis enzymes and secreted pathogenicity factors suggests that artemisinin not only impairs fungal growth but also attenuates virulence at the molecular level.

Taken together, our findings indicate that artemisinin exerts antifungal activity against *S. sclerotiorum* through multiple mechanisms: (i) disruption of cell membrane integrity, (ii) suppression of cell wall–associated and growth-regulatory proteins, and (iii) inhibition of oxalate metabolism and secretion of virulence factors. This multifaceted mode of action may reduce the likelihood of resistance development and enhance its potential as an alternative to conventional fungicides. Nevertheless, the precise molecular targets of artemisinin remain to be elucidated. It is unclear whether artemisinin triggers oxidative stress and metabolic dysfunction by specifically targeting mitochondrial proteins, such as components of the respiratory chain or regulators of membrane potential. Other potential targets—including iron-associated metabolic pathways, cell wall integrity-related proteins, and key virulence regulators—also warrant further investigation. Addressing these mechanistic gaps is essential for understanding the antifungal action of artemisinin and for guiding the rational design of artemisinin-based fungicides.

While this study offers valuable insights into the antifungal activity of artemisinin, several limitations should be acknowledged. Although artemisinin exhibits antifungal activity against *S. sclerotiorum* and shows no apparent phytotoxicity under laboratory conditions, it is important to note that all experiments were conducted in controlled laboratory settings. Whether such efficacy and safety can be consistently achieved under complex field conditions remains to be determined. In addition, the current work focused solely on *S. sclerotiorum*; further investigations involving a broader range of phytopathogenic fungi are needed to clarify the spectrum of activity. Moreover, the relationship between artemisinin and existing chemical fungicides warrants further elucidation. Addressing these aspects in future studies will be instrumental in advancing artemisinin toward practical development as a sustainable crop protection agent, and we will focus on these questions in our subsequent work.

## 4. Materials and Methods

### 4.1. Strains and Culture Conditions

The *S. sclerotiorum* (Lib.) de Bary wild-type strain 1980 (ATCC 18683) was cultured on potato dextrose agar (PDA) medium at 25 °C. PDA plates (prepared with 200 g potato, 20 g dextrose, and 15 g agar per liter of distilled water) were used.

### 4.2. Antifungal Activity Assay

Artemisinin was purchased from MCE (HY-B0094, South Brunswick Township, NJ, USA). PDA medium was prepared containing artemisinin at concentrations of 0.02, 0.06, 0.1, 0.3, and 0.5 mg/mL. The control plates were supplemented with 1% DMSO (*v*/*v*) to match the solvent concentration present in the highest treatment group (0.5 mg/mL artemisinin). Mycelial plugs of *S. sclerotiorum* were placed on PDA plates with or without artemisinin and incubated at 25 °C for three days. Colony diameter was measured at 24 h intervals using a ruler as described by Tremarin et al. (2015) [54], and the growth curve was plotted accordingly. The mycelial growth inhibition rate and EC50 value were subsequently calculated. Based on the artemisinin concentrations and corresponding inhibition rates, the 95% confidence intervals were determined using GraphPad Prism 8.3. The experiment was performed with three replicates.

### 4.3. Scanning Electron Microscopy

*S. sclerotiorum* mycelial plugs were inoculated onto PDA containing varying concentrations of artemisinin. After three days of culture, mycelia were scraped from the plate surface and collected into centrifuge tubes. The samples were suspended in sterile water and centrifuged to form a visible pellet. The supernatant was discarded, and the pellet was gently rinsed with PBS. Following a second centrifugation, the supernatant was removed, and the electron microscopy fixative was added. After fixation, the samples were washed three times (15 min each) with 0.1 M phosphate buffer (PB, pH 7.4), then post-fixed with 1% osmium tetroxide prepared in the same buffer at room temperature in the dark for 1–2 h. Following another three washes with PB (15 min each), the samples were dehydrated through a graded ethanol series (30%, 50%, 70%, 80%, 90%, 95%, 100%, and 100%), each step for 15 min, followed by treatment with isoamyl acetate for 15 min. The samples were placed on coverslips and dried using a critical point dryer (Quorum K850, Laughton, UK). Dried samples were mounted on conductive carbon tape and sputter-coated with gold for approximately 30 s in an ion sputter coater (Hitachi MC1000, Tokyo, Japan). Imaging was performed using a scanning electron microscope (Hitachi SU8100).

### 4.4. Measurement of Cell Membrane Permeability

Twelve 5 mm mycelial plugs were excised from the colony margin using a sterile cutter and inoculated into 250 mL flasks each containing 100 mL of potato dextrose broth (PDB). Cultures were incubated in the dark at 25 °C with shaking (150 rpm) for 3 days. Artemisinin stock solution was then added to achieve final concentrations of 0.02, 0.06, 0.1, 0.3, and 0.5 mg/mL, followed by thorough mixing. Control flasks received 1% DMSO. All flasks were further incubated under the same conditions for 36 h. After treatment, the mycelia were collected on filter paper and washed twice with ultrapure water to remove residual medium. The blotted mycelia (0.1 g) were placed into 15 mL centrifuge tubes containing 3 mL of ultrapure water and vortexed to mix. Electrical conductivity was measured at 0, 10, 30, 60, and 120 min using a conductivity meter. After 120 min, the mycelia were boiled for 5 min, and the final conductivity of the suspension was measured. Relative conductivity of the mycelia was calculated according to Xu et al. (2014) using the formula: Relative conductivity (%) = (Current conductivity/Final conductivity) × 100 [55].

### 4.5. Determination of Malondialdehyde (MDA) Content

MDA content was assessed using the method described by Guo et al. (2020) [56]. Mycelia were treated with artemisinin at concentrations of 0.02, 0.06, 0.1, 0.3, and 0.5 mg/mL and incubated on a shaker for three days. MDA content was measured using an MDA Assay Kit (Solarbio, Beijing, China). Briefly, 0.1 g of mycelium with or without artemisinin treatment was collected, resuspended in 1 mL of extraction solution, and mechanically homogenized in an ice bath. Following centrifugation at 4 °C for 10 min, the supernatant was collected for analysis.

### 4.6. Reactive Oxygen Species (ROS) Assays

The level of ROS was assessed following the method described by Mota et al. (2019) [57]. Briefly, mycelial plugs of *S. sclerotiorum* treated with or without 0.5 mg/mL artemisinin were incubated in 100 mL of PDB medium on a shaker for three days. The mycelia were then stained with 10 μM 2′,7′-dichlorodihydrofluorescein diacetate (DCFH-DA) solution and incubated at 37 °C in the dark for 20 min. After staining, the mycelia were washed three times with PBS and finally observed and photographed using Leica SP8 confocal laser scanning microscope (Leica Microsystems, Wetzlar, Germany).

### 4.7. Determination of Oxalic Acid Content

Oxalic acid content was determined following the method of Kim et al. (2008) with minor modifications [58]. Twelve mycelial plugs (5 mm in diameter) taken from the advancing margin of three-day-old *S. sclerotiorum* colonies were incubated in 250 mL flasks containing 100 mL of PDB with or without different concentrations of artemisinin. After three days of incubation, the culture supernatant was collected. Oxalic acid content was measured using an Oxalic Acid Content Detection Kit (Solarbio, China). Briefly, 50 μL of culture medium was mixed with reagents 1, 2, and 3 as per the manufacturer’s instructions, and the mixture was incubated at room temperature for 20 min. The oxalic acid concentration was determined by measuring the absorbance at 510 nm.

### 4.8. Plant Materials and Growth Conditions

*Arabidopsis thaliana* (Columbia-0) seeds were vernalized in the dark at 4 °C for 2 days. Following surface sterilization with 10% (*v*/*v*) bleach for 10 min and several rinses with sterile distilled water, the seeds were sown on Murashige and Skoog (MS) medium supplemented with 3% sucrose and 0.7% agar. After one week, the seedlings were carefully transplanted to a soil mixture for continued growth. *Brassica napus* ZS11 seeds were directly sown in soil. All plants were cultivated in a growth chamber set at 23 °C under a 12 h light/12 h dark photoperiod, with a light intensity of 100 μmol m^−2^ s^−1^.

### 4.9. Efficacy of Artemisinin on Detached Leaves of Brassica napus and Arabidopsis

Efficacy of artemisinin against *S. sclerotiorum* on detached plant leaves was determined according to Wang et al. (2017) [59]. Fully expanded, healthy leaves of uniform size and color were collected from 4-week-old *Arabidopsis thaliana* and *Brassica napus* plants. An artemisinin solution at a concentration of 0.1 mg/mL was prepared, with 0.2% DMSO serving as the control group. Detached leaves were sprayed with artemisinin solutions or 0.2% DMSO until run-off. After air-drying for 1 h, the leaves were inoculated by placing inverted mycelial plugs taken from the margins of actively growing fungal colonies onto the leaf surface. The inoculated leaves were placed on moist filter paper in trays and incubated in a growth chamber. Leaves were harvested and photographed at 2–3 days post-inoculation. Lesion areas were subsequently quantified using image analysis software and subjected to statistical comparison.

### 4.10. Evaluation of Artemisinin Phytotoxicity on Multiple Plant Species

To evaluate the phytotoxicity of artemisinin, healthy seedlings of *Arabidopsis thaliana*, *Nicotiana benthamiana*, and *Brassica napus*, along with *Brassica napus* plants at the flowering stage, were sprayed with 0.1 mg/mL artemisinin on leaves and flowers, while 0.2% DMSO was used as the control. Plant growth status was observed and photographed two days after treatment.

### 4.11. Protein Extraction and Sample Preparation for Mass Spectrometry

*S. sclerotiorum* mycelial plugs were inoculated onto PDA medium containing 0.05 g/L artemisinin or 0.1% DMSO, respectively. After 3 days of incubation, the mycelia were scraped from the agar surface and washed with sterile water to remove any residual medium. The sample was grounded to powder at low temperature and transferred immediately to the liquid nitrogen pre-chilled centrifuge tube and lysed with lysis buffer, followed by 5 min of ultrasonication on ice. After incubation for 8–15 min at 95 °C and ice-bath for 2 min, the lysate was centrifuged at 12,000× *g* for 15 min at 4 °C, and subsequently the supernatant was added with sufficient iodoacetamide buffer to react for 1 h in the dark. Then samples were completely mixed with 4 times volume of precooled acetone by vortexing and incubated at −20 °C for at least 2 h. Samples were then centrifuged at 12,000× *g* for 15 min at 4 °C and the precipitation was collected. After washing with 1 mL cold acetone, the pellet was completely dissolved by DB buffer (8 M Urea, 100 mM TEAB, pH 8.5). Trypsin was added to the sample, followed by incubation at 37 °C for 4 h. Subsequently, additional trypsin and CaCl_2_ were added, and digestion continued overnight. The digest was acidified with formic acid to pH < 3 and centrifuged at 12,000× *g* for 5 min. The supernatant was slowly loaded onto a C18 desalting column, washed three times with wash buffer (0.1% formic acid, 3% acetonitrile), and then eluted with elution buffer (0.1% formic acid, 70% acetonitrile). The eluates were collected and lyophilized.

### 4.12. LC-MS/MS Analysis-DIA Mode

Mobile phases consisted of solvent A (0.1% formic acid in water) and solvent B (0.1% formic acid in 80% acetonitrile). Lyophilized peptides were reconstituted in 10 μL of solvent A, centrifuged at 14,000× *g* for 20 min at 4 °C, and 200 ng of the supernatant was injected for analysis. Chromatographic separation was performed on a Vanquish Neo nanoUHPLC system equipped with a C18 pre-column (5 mm × 300 μm, 5 μm) and an analytical column (PepMap™ Neo C18, 150 μm × 15 cm, 2 μm, Thermo Fisher, Waltham, MA, USA) maintained at 50 °C. Mass spectrometry was carried out on a Thermo Orbitrap Astral instrument with an Easy-Spray ion source (Thermo Fisher) (spray voltage: 1.9 kV; transfer tube temperature: 290 °C). Data were acquired in data-independent acquisition mode. Full MS scans (*m*/*z* 380–980) were acquired at a resolution of 240,000 (at *m*/*z* 200), with an AGC target of 500% and an isolation window of 2 Th. The number of DIA windows was 300, MS/MS scans were performed with an NCE of 25%, covering *m*/*z* 150–2000 at a resolution of 80,000 (Astral) and a maximum injection time of 3 ms. All raw data files (.raw) were saved for subsequent processing.

### 4.13. Data Analysis

All resulting spectra were searched against the protein database using the DIA-NN library search software (DIA-NN 2.0). The search parameters were set as follows: a mass tolerance of 10 ppm for precursor ions and 0.02 Da for ions fragment. Fixed modifications included alkylation of cysteine and oxidation of methionine. Variable N-terminal modifications considered were acetylation, loss of methionine, and loss of methionine plus acetylation. One missed cleavage was allowed. To ensure high-confidence identifications, the DIA-NN results were filtered to retain only Peptide Spectrum Matches (PSMs) with a confidence level ≥ 99%. Further false discovery rate (FDR) validation was applied at both peptide and protein levels, retaining only those with an FDR ≤ 1%. The identified proteins were subsequently queried against the *S. sclerotiorum* protein database (Uniprot, Swiss-Prot, InterPro, PRIDE) using NCBI BLASTp (BLAST + 2.16.0). Functional grouping of the identified proteins was performed based on InterPro http://www.ebi.ac.uk/interpro (accessed on 5 January 2026). Gene Ontology (GO) enrichment analysis was conducted using the candidate protein set from *S. sclerotiorum*. The differential analysis was performed using a self-developed Perl script by Shanghai Biozeron Biotechnology CO., LTD. *p*-values were generated using the *t*-test model and adjusted using the Benjamini–Hochberg method. The proteins with absolute fold change ≥ 2 and adjusted *p*-value (FDR) < 0.05 were considered significantly different.

### 4.14. Statistical Analysis

All experimental data were obtained from at least three replicates. Significant differences between two samples were determined using a two-tailed Student’s *t*-test in GraphPad Prism 8.3. For comparisons among multiple groups, one-way ANOVA followed by Tukey’s multiple comparison test was applied to the raw data (*n* = 3). Detailed information is provided in the Method Details section and the corresponding figure legends.

## Data Availability

The original contributions presented in this study are included in the article/Supplementary Materials. Further inquiries can be directed to the corresponding authors.

## Supplementary Materials

The following supporting information can be downloaded at: https://www.mdpi.com/article/10.3390/ijms27083422/s1.
