# Supplementary material for: Antifungal Activity and Biochemical Mechanisms of Artemisinin Against the Phytopathogen Sclerotinia sclerotiorum
Source: Int J Mol Sci. 2026 Apr 10;27(8):3422. doi: 10.3390/ijms27083422 (PMC13115749; doi:10.3390/ijms27083422)
Supplement: Supplementary file 1 [file ijms-27-03422-s001.zip › ijms-4191352-supplementary .pdf]

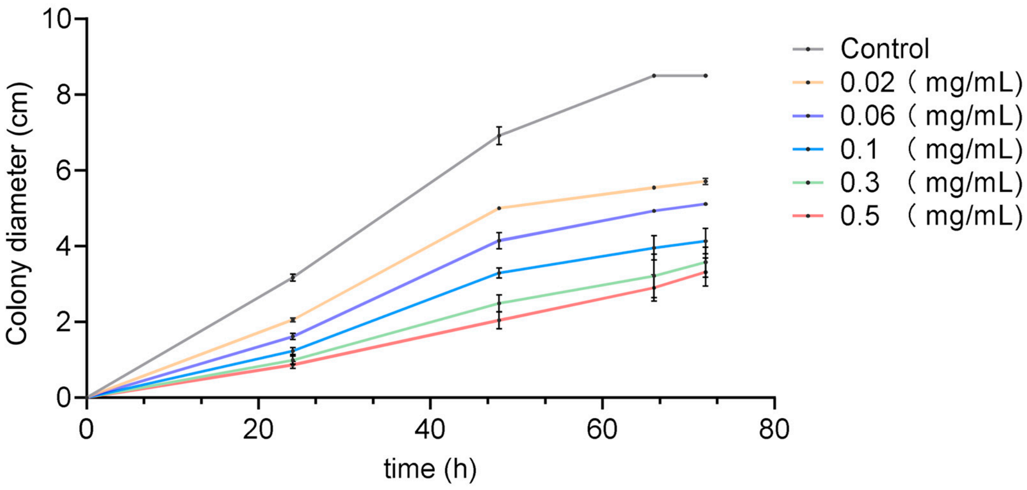

Figure S1. Growth curves of *S. sclerotiorum* treated with different concentrations of artemisinin. The labels on the right indicate the concentrations of artemisinin in the treatment groups, with the unit mg/mL.

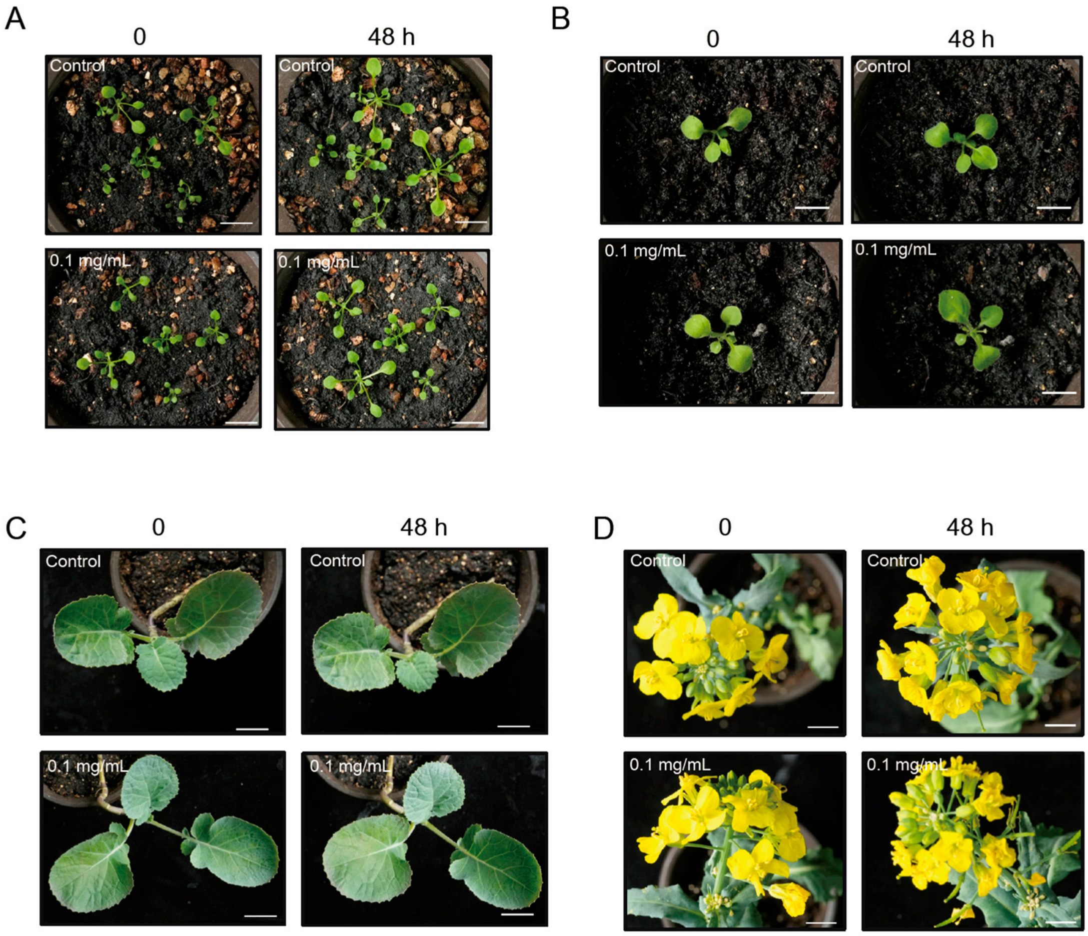

Figure S2. Evaluation of artemisinin phytotoxicity on multiple plant species.

(A) *Arabidopsis thaliana* seedlings were sprayed with 0.1 mg/mL artemisinin or 0.2% DMSO (control). Phenotypes were photographed 2 days after treatment. Scale bar: 1 cm. (B) *Nicotiana benthamiana* seedlings were sprayed with 0.1 mg/mL artemisinin or 0.2% DMSO (control). Phenotypes were photographed 2 days after treatment. Scale bar: 1 cm. **(C)** *Brassica napus* seedlings were sprayed with 0.1 mg/mL artemisinin or 0.2% DMSO (control). Phenotypes were photographed 2 days after treatment. Scale bar: 1 cm. **(D)** *Brassica napus* at the flowering stage were sprayed with 0.1 mg/mL artemisinin or 0.2% DMSO (control). Phenotypes were photographed 2 days after treatment. Scale bar: 1 cm.

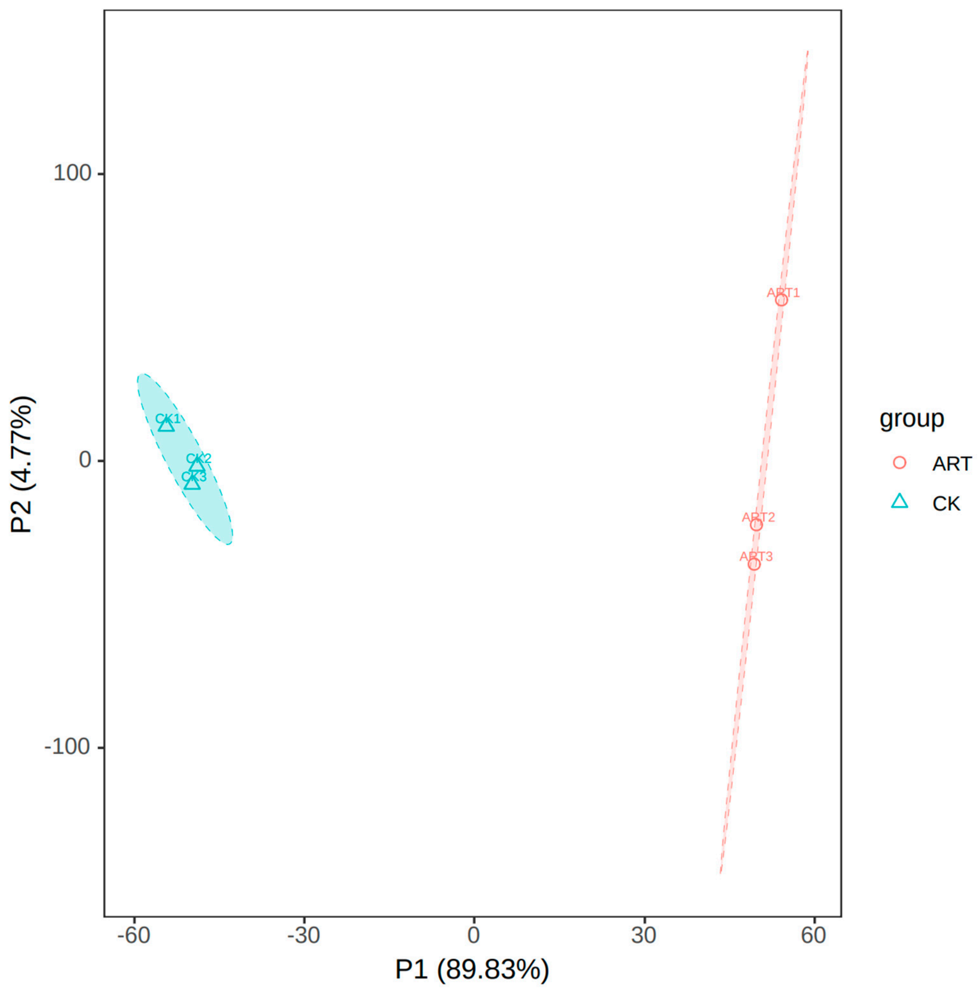

Figure S3. Principal component analysis (PCA) of proteomic profiles.

Principal component analysis (PCA) was conducted to evaluate sample clustering among different treatment groups. Ellipses denote 95% confidence regions. The contribution of each principal component to the total variance is indicated in parentheses. ART1, ART2, and ART3 refer to three biological replicates of the artemisinin-treated group, while CK1, CK2, and CK3 represent three replicates of the control group.

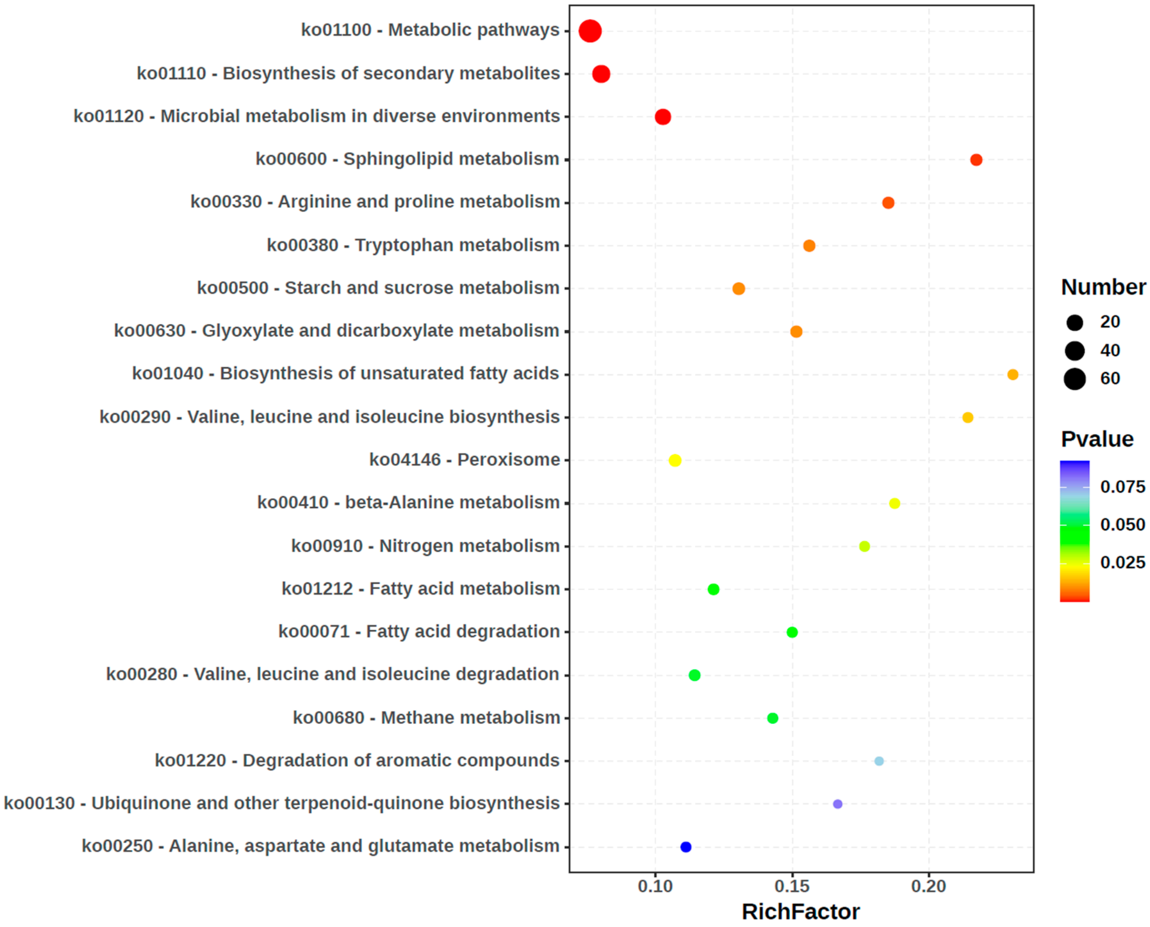

Figure S4. Bubble plot of KEGG pathway enrichment for downregulated proteins in the artemisinin-treated group.

Table S1. Toxicity equation and EC50 of artemisinin against *S. sclerotiorum*

Virulence equation	Correlation coefficient	EC_{50} (mg/mL)	95% confidence limits
Y=60.767+36.228X	0.9766	0.1064	0.09374-0.1446

Table S2. Description of proteins related to oxalate metabolism in Figure 5A.

Accession	Gene name	Description
A0A1D9PUW5	sscle_02g012470	Pyruvate carboxylase
A0A1D9PSY6	sscle_01g006080	Pyruvate carboxylase
A7ESB3	SS1G_08218	Oxaloacetate acetylhydrolase
A7EZM9	SS1G_10796	Cupin type_1 domain_containing protein
A7EMY9	SS1G_06688	Dihydrolipoamide acetyltransferase component of pyruvate dehydrogenase complex
A7EWB7	SS1G_09626	Pyruvate dehydrogenase E1 component subunit alpha
A7F173	SS1G_11343	Pyruvate dehydrogenase E1 component subunit beta
A7E8J7	SS1G_01625	Citrate synthase
A7EAP6	SS1G_02378	ATP citrate synthase
A7EAP7	SS1G_02379	ATP citrate synthase
A7EZ69	SS1G_10635	Aconitate hydratase, mitochondrial
A7F0D0	SS1G_11047	Aconitate hydratase, mitochondrial
A7EB51	SS1G_02537	Isopropylmalate dehydrogenase_like domain_containing protein
A7EFR9	SS1G_04160	Isocitrate dehydrogenase (NAD(+))
A7EHY2	SS1G_04924	Isocitrate dehydrogenase [NADP]
A7F091	SS1G_11008	Dihydrolipoyl dehydrogenase
A7ERA3	SS1G_07857	Succinate__CoA ligase [ADP_forming] subunit beta, mitochondrial
A7ERB0	SS1G_07864	Succinate dehydrogenase [ubiquinone] flavoprotein subunit, mitochondrial
A7EXS9	SS1G_10144	Succinate dehydrogenase assembly factor 2, mitochondrial
A7EIV0	SS1G_05243	fumarate hydratase
A7EU20	SS1G_08827	Malic enzyme
A7F2D2	SS1G_12079	Malic enzyme
A7EHV8	SS1G_04900	Isocitrate lyase
A7EI33	SS1G_04975	Isocitrate lyase
A0A1D9QIF4	sscle_13g094940	Glyoxylate reductase
A7EXV4	SS1G_10169	Alanine__glyoxylate transaminase

Table S3. Description of secreted proteins in Figure 5B.

Accession	Gene name	Description
A7F952	SS1G_14133	FG_GAP repeat protein
A7EB00	SS1G_02486	EF_hand domain_containing protein
A7EFJ4	SS1G_04085	Expansin_like EG45 domain_containing protein
A7EPD5	SS1G_07184	Glycosyl hydrolase family 32 N_terminal domain_containing protein
A7E707	SS1G_01083	alpha_glucosidase
A7EPZ4	SS1G_07393	Pectate lyase superfamily protein domain_containing protein
A7E7I4	SS1G_01262	Asl1_like glycosyl hydrolase catalytic domain_containing protein
A7F6M6	SS1G_13255	beta_glucosidase
A7EP97	SS1G_07146	beta_glucosidase
A7EF50	SS1G_03941	Peptidase A1 domain_containing protein
A7EB09	SS1G_02495	Peroxidase
A7EQN7	SS1G_07639	Acid phosphatase
A7EPU6	SS1G_07345	Hyaluronan_mRNA_binding protein domain_containing protein
A7EIZ1	egd2	Nascent polypeptide_associated complex subunit alpha

Table S4. Description of cell wall proteins in Figure 5B.

Accession	Gene name	Description
A7EE66	SS1G_03606	glucan 1,3_beta_glucosidase
A7E5R7	SS1G_00642	Carbohydrate esterase family 4 protein
A7EGN1	SS1G_04473	Extracellular serine_rich protein
A7EFR1	SS1G_04152	Meiotically up_regulated gene 157 protein
A7F0X0	SS1G_11239	WSC domain_containing protein
A7E572	SS1G_00446	Uncharacterized protein
A7F2W4	SS1G_12262	Allergen Asp f 4
A7EZM9	SS1G_10796	Cupin type_1 domain_containing protein
A7EVB2	SS1G_09270	FAS1 domain_containing protein
A7F7L9	SS1G_13599	Apple domain_containing protein
A7EKS0	SS1G_05917	Apple domain_containing protein
A7EUA0	SS1G_08907	Transglycosylase SLT domain_containing protein
A7EJ44	SS1G_05337	Malate dehydrogenase
A7F952	SS1G_14133	FG_GAP repeat protein
A7ES05	SS1G_08110	Cell wall protein PhiA
A7EUZ2	SS1G_09150	Uncharacterized protein

Table S5. Description of proteins associated with the growth and development of *Sclerotinia sclerotiorum* in Figure 5B.

Accession	Gene name	Description
A7EYL0	SS1G_10426	Protein kinase domain_containing protein
A7F3M0	SS1G_11866	Mitogen_activated protein kinase
A7E910	SS1G_01788	Serine_threonine_protein phosphatase
A7F3R6	SS1G_11912	Necrosis_and ethylene_inducing protein 2
A7F946	SS1G_14127	Gamma_glutamyltranspeptidase
A7F519	SS1G_12694	histidine kinase
A7F952	SS1G_14133	FG_GAP repeat protein
A7EJF2	SS1G_05445	Mitogen_activated protein kinase
A7E8I6	SS1G_01614	Opsin_1
A7EK15	SS1G_05661	Probable metalloreductase AIM14
A7EBU8	SS1G_02784	Catalase
